# Reply on “Association Between Bariatric Surgery and Pelvic Organ Prolapse”

**DOI:** 10.1007/s11695-023-06936-1

**Published:** 2023-11-24

**Authors:** Zhao Tian, Xiuqi Wang, Xiaopeng Hu, Zhijing Sun

**Affiliations:** 1 Department of Obstetrics and Gynecology, Peking Union Medical College Hospital, Peking Union Medical College, National Clinical Research Center for Obstetric & Gynecologic Diseases, Chinese Academy of Medical Sciences, Beijing, China; 2 Department of Structural Heart Disease, National Center for Cardiovascular Disease, China & Fuwai Hospital, Chinese Academy of Medical Sciences & Peking Union Medical College, Beijing, China

Dear Editor,

We are writing in response to the letter titled “Association between bariatric surgery and pelvic organ prolapse” which provided valuable comments on our previous article. Firstly, we sincerely appreciate the author’s attention to our study and the insightful comments raised. As mentioned by the author, there was substantial heterogeneity in Fig. 1 of our previous study, and subsequent predictive analysis indicated the instability in the results. We acknowledge this limitation in our previous study, and in fact, we have explicitly stated this limitation in the article as well.

Given the significant heterogeneity detected in Fig. 1 in our previous study, we employed a random-effects model for analysis and further conducted subgroup analyses. Unfortunately, due to the limited number of included studies, we were unable to identify the source of heterogeneity through these subgroup analyses. However, it is noteworthy that we observed indications of bariatric surgery potentially improving symptoms of pelvic organ prolapse (POP) during both short-term (3–6 months) and long-term (≥ 12 months) follow-ups.

Currently, the prevalence of obesity and pelvic floor disorders are increasing, adversely affecting women's quality of life. Elucidating the relationship between bariatric surgery and improvements in POP may offer novel insights for the prevention and management of this condition. However, as emphasized by Professor Hung and our previous research, additional high-quality studies are imperative to strengthen and validate our findings in future follow-up investigations.

We would like to express our gratitude once again for Professor Hung’s interest in our research, and we also appreciate the opportunity to address these concerns.

